# Obstructive sleep apnea in obese minipigs

**DOI:** 10.15761/jts.1000374

**Published:** 2020-01-27

**Authors:** Meng-Zhao Deng, Mohamed Y Abdelfattah, Michael C Baldwin, Edward M Weaver, Zi-Jun Liu

**Affiliations:** 1 Department Orthodontics, University of Washington, Seattle, USA; 2 The First Affiliated Hospital of Shenzhen University, Health Science Center, Shenzhen, China; 3 Department Oral Biology, Beni-Suef University, Beni-Suef, Egypt; 4 Department Oral Health Sciences, University of Washington, Seattle, USA; 5 Department Otolaryngology/Head & Neck Surgery, University of Washington, Surgery Service, Seattle Veterans Affairs Medical Center, Seattle, USA

**Keywords:** obstructive sleep apnea, obesity, sleep stages, animal models, swine

## Abstract

**Objective:**

Obesity has reached epidemic proportions and is a strong risk factor for obstructive sleep apnea (OSA). However, the underlying mechanisms are poorly understood and current treatment strategies for OSA and obesity have critical limitations. Thus, establishment of an obesity-related large animal model with spontaneous OSA is imperative.

**Materials and methods:**

Natural and sedated sleep were monitored and characterized in 4 obese (body mass index - BMI>48) and 3 non-obese (BMI<40) minipigs. These minipigs were instrumented with the BioRadio system under sedation for the wireless recording of respiratory airflow, snoring, abdominal and chest respiratory movements, electroencephalogram, electrooclulogram, electromyogram, and oxygen saturation. After instrumentation, the minipigs were placed in a dark room with a remote night-vision camera for monitoring all behaviors. Wakefulness and different sleep stages were classified, and episodes of apneas and/or hypopneas were identified during natural and/or sedated sleep.

**Results:**

No hypopnea episodes were observed in two of the non-obese minipigs, but one non-obese minipig had 5 hypopnea events. Heavy snoring and 27-58 apnea and/or hypopnea episodes were identified in all 4 obese minipigs. Most of these episodes occurred in the rapid eye movement stage during natural sleep and/or sedated sleep in Yucatan minipigs.

**Conclusions:**

Obese minipigs can experience naturally occurring OSA, thus are an ideal large animal model for obese-related OSA studies.

## Introduction

Obstructive sleep apnea (OSA) affects up to 20% of the population with significant morbidity and mortality, and the prevalence is increasing as obesity reaches epidemic proportions. A number of studies have indicated that obesity is a strong risk factor for OSA and changes in body weight relates to OSA severity [1]. Since obesity and OSA share some common mechanisms such as inflammatory activation, oxidative stress, and increased sympathetic activation, they may interact and potentiate with each other [2]. The efficacy of various treatments for OSA, including mechanical and surgical applications, experimental neuromuscular stimulation, and pharmacological interventions, are still in debate, and each treatment strategy has its critical limitations. In addition, the evaluations of treatment effectiveness are greatly limited by constraints in measuring anatomic and functional variables in humans. Therefore, a validated and characterized large animal model is needed.

The pig is an ideal animal model for OSA study. They are similar to humans in body size and airway structures, and have similar sleep stages and EEG activity as humans. Same as in humans, sleep stages of the pig include rapid eye movement (REM) and non-rapid eye movement (NREM). The REM sleep stage is characterized by desynchronized electroencephalogram (EEG), closed eyes and burst of large-amplitude eye movements in the EOG, and the NREM is characterized by closed and very slow moving eyes in EOG and almost synchronous activity with 2-12 Hz slow-wave rhythms in electrooclulogram (EOG). The NREM has been also named slow-wave sleep [3-5]. Although OSA can be induced in monkeys [6], minipigs [4], dogs [7], rabbits [8], cats [9], rats [10], and mice [11], only spontaneous OSA is acceptable for studying the mechanisms of OSA and direct effects of treatment modalities. Spontaneous OSA and sleep-related breathing disorders have been reported in obese Yucatan and Vietnamese pot-bellied minipigs [4,12]. However, there are several critical limitations in these two minipig studies: 1) extremely small sample size (n=1-2); 2) the lack of standardized instrumentation for physiological variables; and 3) the respiration and sleep monitoring devices were bulky and wired which placed the animals in a restrained condition. To the best of our knowledge and ample experiences working with minipigs, such instrumentation is not repeatable in a freely moving minipig, and thus unlikely to collect reliable respiration and sleep data from natural sleep. Therefore, the purposes of the present study are: 1) develop an easier, standardized, and wireless instrumentation to allow real-time recording of physiological variables from the natural sleep in a free-moving minipig; and 2) establish an obesity-related large animal model by validating and characterizing spontaneous OSA.

## Materials and methods

### Animals

**Figure 1.**
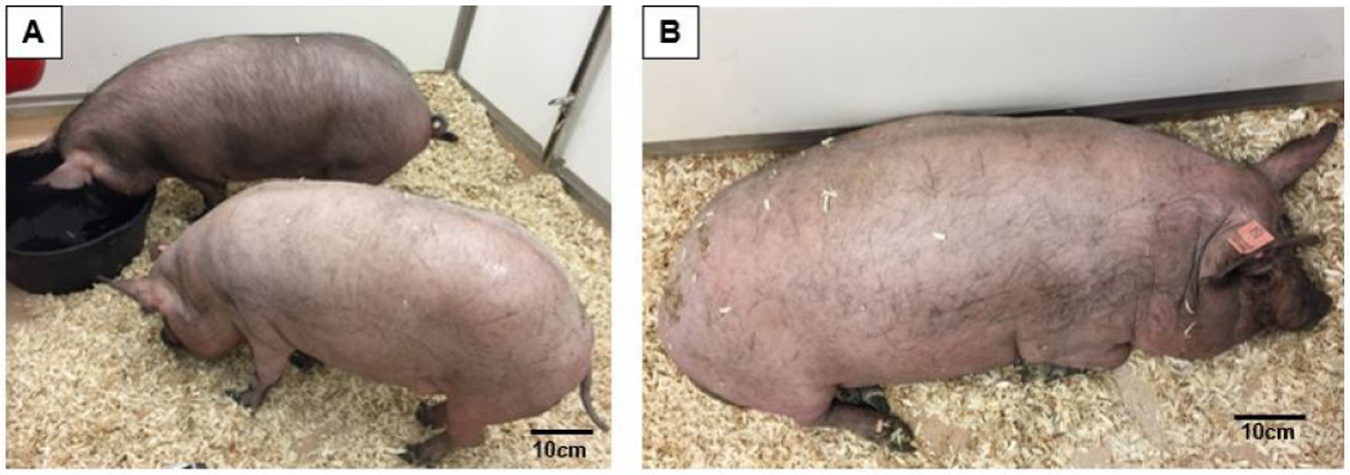
(A) Yucatan minipigs with BMI < 40 (non-obese control minipigs #970 and #981). (B) Yucatan minipig with BMI > 50 (obese minipig #954). BMI: Body mass index

Five 8-11-month-old Yucatan (S&S Farms, CA) and two 6.5-year-old Panepinto (Panepinto & Associates, CL) minipigs were used for this study. Of 5 Yucatan minipigs, 3 were non-obese controls and 2 were obese (Figure 1) and both Panepinto minipigs were obese. The physical parameters of these minipigs were summarized in Table 1. Per the guideline of the Center for Disease Control and Prevention (CDC) of the US, the values of BMI for normal, overweight, and obese adults are defined as 18-25, 26-30, and over 30, respectively. Based upon the growth database of Yucatan minipigs from the Sinclair BioResources, MO, a large minipig vendor and research institute, the BMI for a Yucatan adult minipig is 28-30. Assuming the BMI 31-39 could be considered as overweight, a BMI larger than 40 was determined to be the cutoff line to distinguish obesity from normal minipigs in the present study. Because minipig breeds have an inherent tendency to obesity and gain fat readily under an appropriate feeding regime, obese minipigs for the present study were specifically raised by ad-lib feeding for at least three months by the vendors before delivery. Upon arrival, the minipigs’ feeding regime was maintained, and to acclimate them to the recording devices, each pig was trained daily for a week by wearing a jacket which would be used for securing the recording equipment during sleep monitoring sessions. In addition, a daily reverse lighting schedule (6 PM – 6 AM) was applied for inducing natural sleep in the dark during daytime. The Institutional Animal Care and Use Committee of the University of Washington approved all procedures (Protocol# 3393-04).

### Instrumentation

**Figure 2.**
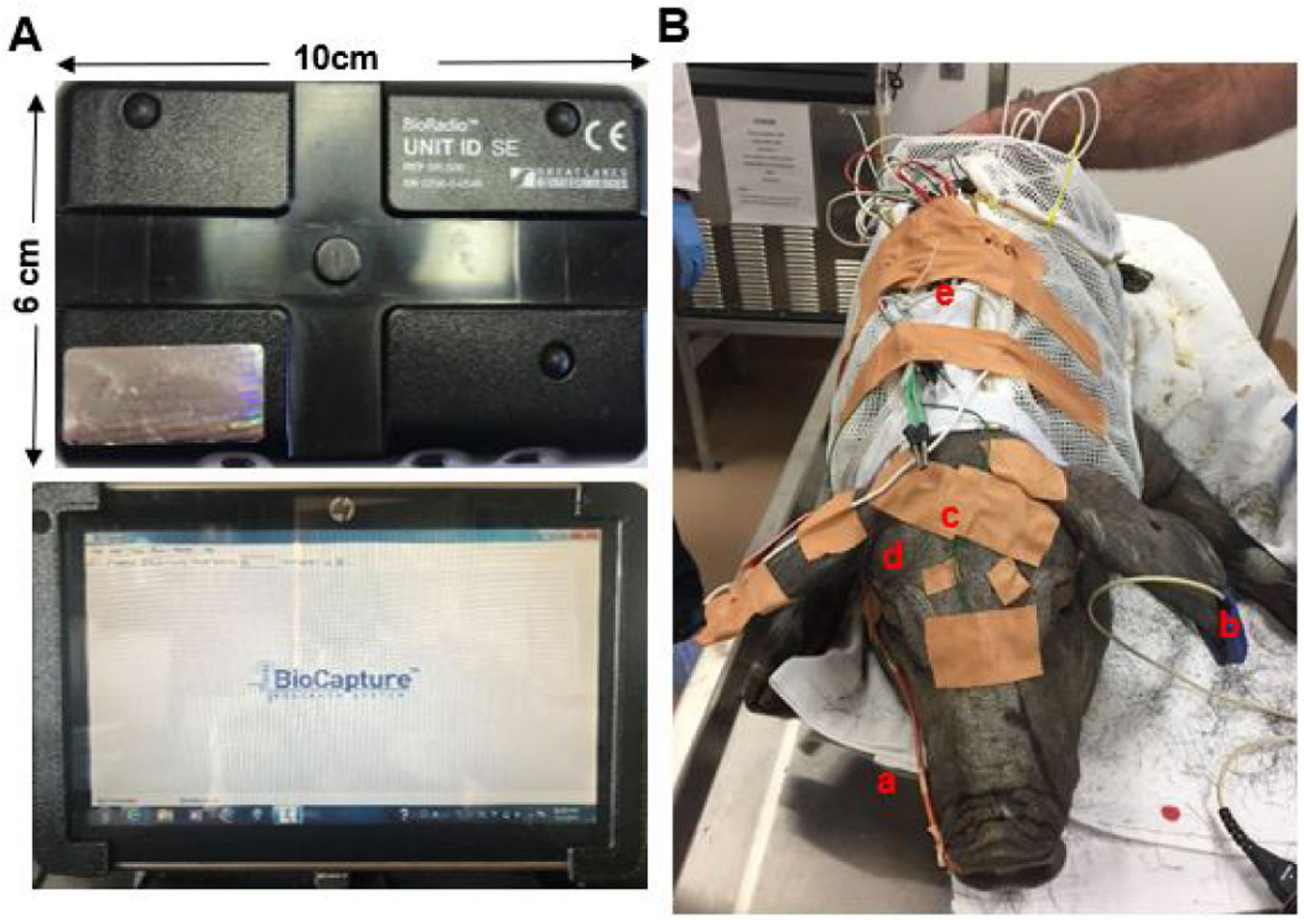
(A) BioRadio system. Upper: BioRadio box: a wearable device with programmable channels for recording and transmitting combinations of physiological signals; Bottom: wireless recording laptop with real-time signal tracing. (B) Two wireless BioRadio boxes were instrumented under sedation. a: nasal catheter for respiration; b: ear clip for oxygen saturation; c: wire electrodes for EEG d: wire electrodes for EOG; e: two BioRadio boxes placed in the pocket of a customized minipig jacket

Respiratory airflow and movements, oxygen saturation, and multi-physiological variables were collected using BioRadio Wireless Recording System (Great Lakes Neuro-Technologies, OH). This is a wearable device with programmable channels for recording and transmitting combinations of multiple physiological signals, and real-time signal tracing can be acquired wirelessly by using its BioCapture software (Figure 2A).

After the combination of xylazine (4 mg/Kg), midazolam (0.5 mg/Kg) and butorphanol (0.3 mg/Kg) was injected I.M. to induce sedated sleep, the following procedures were performed: 1) a custom-made jacket with a pocket on the back for holding the recording device and worn during the first week of training was placed on the pig; 2) a modified French red rubber nasal catheter (#8-10) was inserted into the right nasal cavity with the depth of 5-6 cm. This catheter was sutured to the nearby skin of the snout for stabilization and connected to a respiratory sensor to record the airflow and snoring; 3) two elastic belts were placed around the abdomen and chest, then connected to their sensors to record respiratory movements; 4) an ear clip oximeter was clamped and taped to record oxygen saturation and pulse rate; 5) two mono-polar wire electrodes were inserted into the sites approximating C3 and C4 in humans through the scalp to reach the surface of the skull for electroencephalogram (EEG) with references electrodes on the forehead; 6) the two same wire electrodes were inserted into both lateral canthi for electrooclulogram (EOG); and 7) a bipolar wire electrode with the separation of 2 mm was inserted into the right pharyngeal middle constrictor (MPC) for electromyography (EMG) with a ground electrode placed in the back ear (Figure 2B). The MPC was accessed by inserting the guiding spinal needle 30 mm perpendicularly at the middle point of the posterior border of the mandibular ramus. Resistance was felt when the needle tip reached the hyoid, then the needle was withdrawn slightly to hook the bared tips of the electrodes in the muscle. All these electrodes were nickel–chromium fine-wire (California Fine Wire Co. Grover Beach, CA) with 1 mm bared tip, and connected with each individual lead. Finally, all device cables and electrode leads were stabilized using surgical tape and twist ties, and connected to the two BioRadio recording boxes: box A was connected to the sensors of airflow/snoring, two belts, and oximeter, and box B was connected to the leads of EEG, EOG, and EMG.

### Sleep monitoring

**Figure 3.**
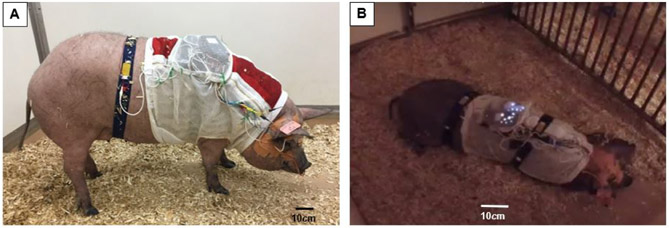
(A) The minipig #954 with full instrumentation awoke from sedation and was in a freely moving status. (B) The same minipig fell into natural asleep after waking up from sedation. The flashing dots on the back of the minipig are from the two BioRadio boxes

After the instrumentation, the minipig was sent back to the pen, and Yohimbine (0.125-1.0 mg/kg) was injected I.M. as a reverse agent for xylazine. While alone and in the dark, the minipig’s behaviors, and the status of wakefulness and sleep positions were closely monitored and noted outside of the pen via a remote night-vision camera (Yi Technologies Inc. Shanghai, China). The minipig usually remained in sedated sleep for about 20-30 minutes, and gradually woke up in a freely moving status for about 15-20 minutes (Figure 3A), then fell asleep again (Figure 3B). The real-time recordings (8-bit resolution with a sampling rate of 256 Hz) was started after the reversal agent was injected, and ended when the pig woke up from one or more natural sleep sessions lasting longer than 0.5 hours, or did not fall asleep for more than 2 hours after waking up from sedated sleep. The total recording times for each minipig varied from 1.5 to 4 hours. Of 4 obese minipigs, 2 (#930 and #10) were recorded a second time 4-5 days after the first one. Data from these two recordings in the same minipigs were compared for testing the reproducibility of the sleep monitoring methods. After sleep monitoring, all instrumented devices were removed, and the minipig was housed for further study.

### Data analysis and statistics

All recorded videoclips were reviewed and analyzed together with physiological variables using BioCapture/VivoSense software provided by the same vendor. The band-pass filters for EEG were set 0.08 to 40 Hz, and the lower cut off for EOG and EMG was 20 Hz. As done in other studies [3,5,13], both natural and sedated sleep were divided into the three stages, i.e., wakefulness, rapid, and non-rapid eye movement sleep (REM and NREM) according to their behavior and the presentations of EEG/EOG/EMG.

Because the sleep or resting respiratory rates in these minipigs were much faster (25-30/min) than that of adult humans (12-18/min) [14], the length of each apnea or hypopnea episode was defined as 5 seconds or longer, rather than 10 seconds or longer as applied clinically for adult sleep apnea and hypopnea episodes, i.e., apnea/hypopnea index (AHI) defined as the number of apneas and/or hypopnea episodes per hour of sleep. Apnea episodes were defined as>90% decrease in airflow lasting 5 seconds or longer, and hypopnea episodes were defined as>30% decrease in airflow or loss of plateau on the tidal volume with a decrease in oxygen saturation for 5 seconds or longer. Because sleep apnea and hypopnea can be categorized as obstructive, central, or mixed, paradoxical movement of the abdomen and chest from the two belts were used to identify the category of sleep apnea/hypopnea.

Due to the small sample size and limited successful recordings, descriptive statistics were applied to each individual animal to characterize the sleep stages and apnea/hypopnea episodes, along with physical and respiratory parameters, and to demonstrate the proof of concept of verifying and quantifying OSA. The non-parametric Kruskal-Wallis test was used to examine the significant difference of AHI in non-obese control and obese minipigs. The significant level was set as p<0.05.

## Results

### Reproducibility of the methods

The respiratory parameters and AHI of the same obese minipig were compared between the two monitoring sessions of natural sleep recorded on different days (4-5 days apart). The respiratory rates were 23-30 vs 24-31 per minute and the tidal volumes were 200/120 vs 300/200 milliliters in the first and second recordings, respectively, and the range of AHI was 32-34. These numbers demonstrate the methods for natural sleep monitoring is reproducible.

### Sleeping behaviors and positions

**Figure 4.**
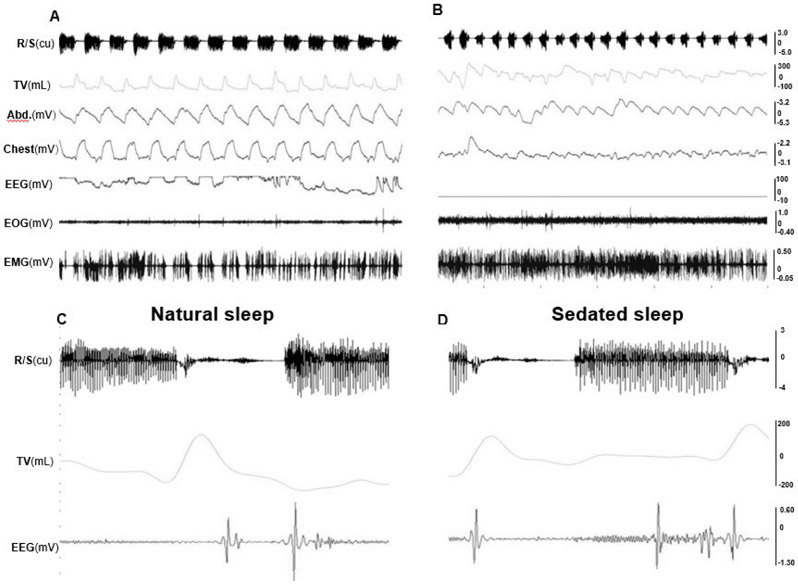
Raw tracings of physiological variables during natural and sedated sleep in the obese minipig #10. (A) Natural sleep (30 seconds). (B) Sedated sleep (30 seconds). (C) respiration/snoring, tidal volume, and EEG during natural sleep (5 seconds). (D) respiration/snoring, tidal volume, and EEG during sedated sleep (5 seconds). Please note that C and D are not the zoomed tracings of A and B, but from the same recording session in the minipig #10. Res/Snore: Respiration/Snoring; TV: Tidal volume; Abd.: Abdominal movement; Chest: Chest movement; EEG: Electroencephalogram of the sites C3 and C4; EOG: Electrooculogram; EMG: Electromyogram of right pharyngeal middle constrictor. Please note that tracings of peripheral capillary oxygen saturation (SpO2) are not listed, and the EEG in B is flat, because these recordings were not successful in this specific session

After waking up from sedated sleep for about 15-20 minutes, two Yucatan and one Panepinto obese minipigs fell back to sleep for more than an hour. Due to the use of a reverse agent for xylazine, this type of sleep is unlikely to have occurred from the lingering effect of the sedative. Therefore, it was defined as natural sleep in the present study. These natural sleep sessions were interrupted by several brief periods of wakefulness lasting a few minutes. However, 3 non-obese Yucatan minipigs and one obese Panepinto (#7) minipig did not fall back to natural sleep more than 2 hours after waking up from sedated sleep. During either sedated or natural sleep, several position changes were observed but side-positioned sleeping was dominant for both non-obese and obese minipigs. Heavy snoring was presented in all 4 obese minipigs during both sedated and natural sleep, whereas only light snoring was found in one of 3 non-obese minipigs (Pig#716). The comparisons of recorded physiological variables between sedated and natural sleep confirmed that their snoring, respiratory movements of abdomen and chest, and heart beat rates were similar (Figures 4A and 4B) with the same traits of EEG (Figures 4C and 4D), but both inspired and expired tidal volumes during natural sleep were larger than those of sedated sleep, and the respiratory rates of both natural and sedated sleep were lower in Panepinto than those of Yucatan minipigs (Table 2).

### Characterization of sleep stages

**Figure 5.**
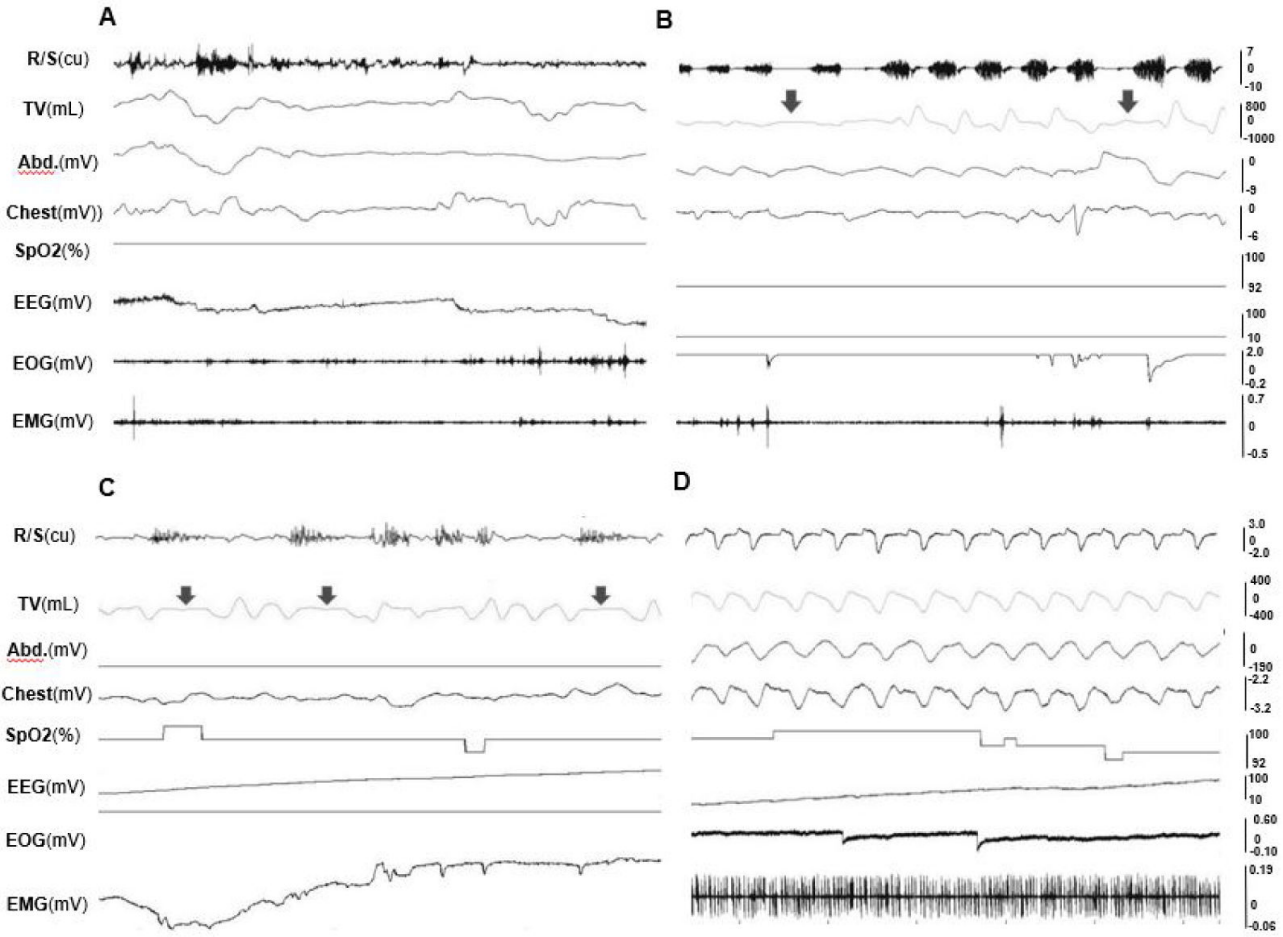
Raw tracings of physiological variables during wakefulness and sleep stages (30 seconds). (A) wakefulness (obese #10). (B) REM sleep stage; (C) NREM sleep stage (obese #954); (D) REM sleep stage (non-obese #716). Arrows indicate hypopnea episodes. Please note that since no snoring occurred in the non-obese minipig#716, the R/S tracing in D shows the respiratory airflow waves. See Figure 4 for all captions

**Figure 6.**
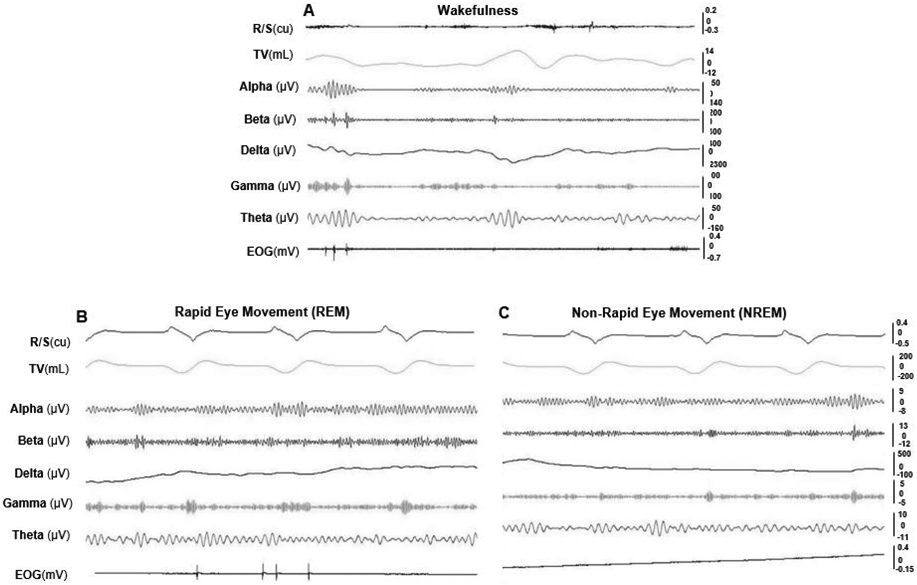
VivoSense processed EEG and EOG wave patterns of wakefulness and sleep stages in an obese minipig #970 (10 seconds). (A) wakefulness stage (B) REM sleep stage. (C) NREM sleep stage. See Figure 4 for all captions

During wakefulness, minipigs were alert or moved around, and exhibited lower amplitude and higher frequency EEG with more EMG activity, large and frequent eye movement, and irregular respiratory cycles and heart beats. In REM sleep, the EEG showed higher frequency, but the amplitude was lower, along with decreased EMG activity. EOG captured rapid and random movements of the eyes, and respiration was regular and at its lowest rate. More apnea and/or hypopnea episodes occurred during REM sleep. In NREM sleep, EEG waves showed a relatively slower activity but higher amplitude, and almost no movement of the eyes in EOG. Figure 5 shows the typical raw tracings of physiological variables during wakefulness (A), REM (B), and NREM (C) stages in obese minipigs, and REM sleep recording in a non-obese minipig (D). Figure 6 further shows the typical presentations of EEG and EOG wave patterns processed by the VivoSence software during wakefulness, REM and RREM sleep stages in an obese minipig. During wakefulness, there were more appearances of beta and gamma waves and less active theta waves in EEG, and irregular eye movement in EOG (A). In the REM stage, EEG presented a higher frequency of alpha waves with lower amplitude along with a higher frequency of beta waves, and intermittent eye movement was seen in EOG (B). In the NREM stage, the EEG was featured by more synchronized beta and gamma waves but slower alpha waves, and the eye movement diminished in EOG (C).

### Apnea and hypopnea episodes

Since the signals of respiratory movements from abdominal and chest belts were always seen during all recordings, no central or mixed apnea was identified. Of 3 non-obese Yucatan minipigs, only one (#716) experienced hypopnea episodes (AHI=5). As shown in Table 3, during sedated sleep, both REM and NREM AHIs were all significantly greater in obese than non-obese minipigs (p<0.05), and the total and NERM AHIs were significantly higher in obese Panepinto than obese Yucatan minipigs (p<0.05). Furthermore, AHIs were more often observed during REM than NERM stages during natural sleep in both obese Yucatan and Panepinto minipigs, but this was not the case during sedated sleep for older Panepinto minipigs (Table 3). The ranges of oxygen saturation were wider in obese (85-100%) than non-obese (93-100%) minipigs during both natural and sedated sleep, and no difference of oxygen saturation between natural and sedated sleep. In obese minipigs, irregular breaths and absent/decreased airflow were more often seen in REM than NREM stages, followed by a brief arousal. Interestingly, apnea and hypopnea episodes lasting 5-10 seconds were more often seen during sedated sleep, whereas those lasting longer than 10 seconds occurred more often during natural sleep.

## Discussion

### Merits and limitations of the methodology

The BioRadio system was used for this study, which provided the following advantages: 1) small, wireless, and well tolerable by large, freely moving non-obese and obese minipigs; 2) easy and minimally invasive instrumentation (only with the insertions of 0.05 mm wire electrodes) allowing a full setup of sleep monitoring recordings, similar to a clinical polysomnography (PSG); 3) clear identification of sleep stages and respiratory disturbances. However, there are also several limitations: 1) some respiratory parameters, such as airflow pressure and peak inspiratory and expiratory pressures (PIP and PEP), were not available; 2) oxygen saturation sometimes went above 100% for unclear reasons; 3) some recordings, especially EMG from the MPC, were sometimes disturbed when sleeping on the side position; 4) the loss of some signals occurred due to the dislodgement of leading cables or wire electrodes. Nevertheless, this methodology is a feasible approach to identify sleep stages and characterize sleep apnea and hypopnea episodes in a large animal model. The comparisons of the respiratory parameters and AHI during natural sleep sessions from different days in the same minipigs further demonstrate that this methodology is reproducible for sleep monitoring in a freely moving large animal.

### An appropriate large animal model for OSA study

Even though the clinical features of OSA have been well characterized, its pathogenesis and pathophysiology are poorly understood, and each treatment strategy has critical limitations. Surgical intervention and tongue nerve stimulation are invasive and have unpredictable success and an unclear mechanism of effect. Conservative treatments, such as CPAP, oral appliances and physical therapy have either a low tolerant rate by patients or controversial outcomes. Therefore, there is an urgent need to establish a suitable large animal model for the study of OSA mechanism and testing of treatment strategies, including potentially curable adipose tissue reduction in the upper airway and pharmaceutical therapy. An ideal animal model should be easy to manipulate, exhibit naturally occurring OSA, and be large enough for the instrumentation. English bulldogs were reported to have spontaneous OSA episodes, but its mechanism is unique since this type of breed has severely abnormal craniofacial anatomy with a narrow oropharynx and relatively long soft palate [7]. The obese Zucker rats had more collapsible upper airway compared with lean controls as judged by more positive critical closing pressures [15]. However, in addition to the huge difference from human upper airway structures, the size of rats is not suitable for a full instrumentation of sleep monitoring. Our choice to study obese minipigs was based upon the following facts: 1) spontaneous OSA or hypopnea episodes have been reported in 1-2 obese minipigs, albeit not well validated and characterized [4,12]; 2) the upper airway of the minipig is comparable to that of humans, both showing a similar microstructural layered architecture composed of the same tissue types [16]; 3) the minipig’s tongue resembles that of humans in both shape and size; and the tongue base strongly influences the upper airway through its contact with the soft palate [17]; 4) the minipig provides adequate size for instrumentation and therapeutic intervention; 5) the time course of epiglottic movement in minipigs is similar to that of humans [18]; and 6) the size and shape of the minipig’s hyoid conform with those of humans [19].

In the present study, the episodes of apnea or hypopnea usually lasted 5-10 s, differing from humans who experience apneas or hypopneas for longer than 10 s. This fact confirms that the actual length of apnea or hypopnea is proportional to the respiration rate. However, compared to Yucatan minipigs, the aged and heavy-weighted Panepinto minipigs (Table 1) presented much lower respiratory rates and higher tidal volume during both natural and sedated sleep as compared with young-aged Yucatan minipigs (Table 2). These facts indicate that like humans, the respiratory rate decreases with age, and the tidal volume is closely related to the body size and weight in pigs.

A recent human imaging study revealed that OSA patients with obesity are associated with greater fat deposition in the tongue base as compared with BMI matched controls [20]. This compelling finding suggests a new curable treatment approach by reducing fat tissue in the tongue base. Clearly, such invasive intervention should be tested in an appropriate animal model first. Therefore, an obese animal model with spontaneous OSA and greater fat deposition in the tongue base and other oropharyngeal structures is of critical value. The comparisons of fat tissue in the tongue base, soft palate, and pharyngeal walls between these obese-OSA and non-obese minipigs is an ongoing study in our group [21].

### Natural and sedated sleep

Sedated and natural sleep are the two states of unconsciousness with considerable physiological similarities [22]. Studies have shown that oral administration of sedation drugs has no effect on ventilatory response [23], and the confounding effect of light anesthesia on respiration is minor [24]. In addition, studies also found that EEG findings appear identical during sedated and natural sleep in horses [22], and patients with anatomically compromised airways tend to have an obstructed airway in either state because depressant effects on muscle activation and ventilatory drive are shown in both states [25]. However, patients with sleep breathing disorders may have more severe symptoms since arousal responses are depressed during sedation [25]. Therefore, it is reasonable to assume that the 3 non-obese minipigs might not have OSA or hypopnea during natural sleep as they did not present OSA or hypopnea during sedated sleep.

The respiration rate and lower tidal volume were higher in sedated than natural sleep in 3 obese/OSA minipigs (Table 2), and more higher respiratory rate was also seen under intubated-anesthesia during terminal experiments in these minipigs (60-65/min) [26]. These facts may explain why apnea/hypopnea episodes lasting 5-10s occurred more often during sedated sleep while the episodes longer than 10s were mostly seen during natural sleep. Collectively, although certain differences exist between sedated and natural sleep, it appears that multiple obstructive apnea and/or hypopnea episodes identified in either sleep state reflect the real OSA, thus the obese minipig is an ideal animal model for OSA study.

In obese/OSA minipigs, more apnea or hypopnea episodes were found in REM than NREM stages during natural sleep, and the ratios of REM/NREM related apneas and hypopnea episodes was about 2. This REM-predominant OSA mimics a common pattern seen in both pigs [4] and humans [27]. Even though the clinical significance of REM only OSA remains controversial, studies have shown that OSA-induced metabolic perturbation, including atherogenesis, hypertension, cardiovascular disease, and abnormal glucose metabolism, are more strongly related to obstructive respiratory events during REM than NREM sleep [27]. Interestingly, during sedated sleep, these REM-predominant apnea/hypopnea episodes were only seen in young obese and non-obese Yucatan, but not obvious in older obese Panepinto minipigs. In addition, AHIs were significantly greater in these older Panepinto than young Yucatan minipigs (Table 3). These difference in OSA characteristics between these two minipig breeds may be related to their ages and/or breed variations.

### Obesity and OSA

OSA is strongly associated with obesity, and the prevalence of OSA has been substantially increased over the past two decades due to the obesity epidemic. More than 60% of OSA patients are obese [1]. There is a complex relationship between obesity and OSA. Obesity may accumulate adipose tissue around the pharyngeal structures (i.e., tongue base, soft palate, and pharyngeal wall), resulting in a narrowed upper airway, altered tissue properties, increased critical pressure, reduced resting lung volume, and ultimately hypoxemia and alteration in metabolic hormones [28]. On the other hand, OSA may also accelerate weight gain, as sleep fragmentation often seen in OSA is associated with decreased leptin and increased ghrelin levels [29]. Disturbances in energy metabolism and insulin resistance have been seen in OSA patients [30]. Furthermore, presentations of adipose tissue dysfunction resulting from OSA shows a striking resemblance to those induced by obesity [31]. Therefore, OSA and obesity share similar pathophysiological mechanisms and may interact with and potentiate each other. Studying the direct interactions between OSA and obesity in humans could be difficult, as the interventions to alter OSA and/or obesity are demanding, complex, and invasive. The present study provides a suitable large animal model to investigate the respective roles and interactions of OSA and obesity.

## Conclusions

The present study demonstrated that obese minipigs present naturally occurring OSA, thus are an ideal large animal model for obese-related OSA study. The BioRadio system used in the present study is a useful tool for monitoring sleep and characterizing sleep events in freely moving minipigs.

## Figures and Tables

**Table 1.** Physical characteristics of minipigs

Pig No.	Age	Sex	BW (Kg)	BL (cm)	BMI (Kg/m^2^)	NC (cm)
Obese Yucatan						
930	8.5 M	M	68	115	51.42	82
954	8.5 M	F	71	119	50.13	77
**Obese Panepinto**						
7	6.5 Y	M	85.6	132	48.75	82
10	6.5 Y	M	104.1	132	59.30	94
**Non-obese Yucatan**						
716	8.0 M	F	45.9	110	37.93	61
970	9.5 M	M	55	120	38.19	66
981	11.0 M	F	55.1	119	38.91	74

**BW:** body weight; **BL:** body length; measured from the tip of snout to the base of tail. **BMI**: body mass index, calculated at body weight (Kg) /body length(m)^2^. **NC:** neck circumference, measured at the location of thyroid cartilage.

**Table 2.** Comparisons between natural and sedated sleep

	RR (minute)		TV (mL)		HR (minute)	
Pig No.	N	S	N	S	N	S
**Yucatan**						
930	20-24	23-31	700/300	300/300	75-81	80-85
954	20-25	25-35	900/650	700/400	79-81	78-82
**Panepinto**						
7	n/a	12-19	n/a	1300/700	n/a	n/a
10	15-21	19-26	1,900/1,500	1,800/1,300	N/A	77-84
**Non-obese Yucatan**						
716	n/a	12-28	n/a	300/300	n/a	63-86
970	n/a	27-41	n/a	200/300	n/a	69-91
981	n/a	14-23	n/a	400/400	n/a	45-57

**RR:** Respiratory rate; **TV:** Tidal volume, inspired TV/expired TV; **HR:** Heart rate; **N & S:** Natural & Sedated sleep. **n/a:** not available.

**Table 3.** Comparison of AHI during natural and sedated sleeps

	Natural			Sedated		
Pig No.	Total	REM	NREM	Total	REM	NREM
**Obese Yucatan**						
930	32	19	13	30	17	13
954	35	35	0	31	23	8
**Obese Panepinto**						
7	n/a	n/a	n/a	59	27	32
10	28	23	5	53	17	36
**Non-obese Yucatan**						
716	n/a	n/a	n/a	5	5	0
970	n/a	n/a	n/a	0	0	0
981	n/a	n/a	n/a	0	0	0

**AHI:** Apnea and hypopnea episodes per sleep hour. **REM & NREM:** rapid & non-rapid eye movement stages, respectively.

**n/a:** not available.
